# Determinants of joint effusion in tarsocrural osteochondrosis of yearling Standardbred horses

**DOI:** 10.3389/fvets.2024.1389798

**Published:** 2024-07-24

**Authors:** Andrea Bertuglia, Marcello Pallante, Eleonora Pagliara, Daniela Valle, Lara Bergamini, Enrico Bollo, Michela Bullone, Barbara Riccio

**Affiliations:** ^1^Dipartimento di Scienze Veterinarie, Università di Torino, Turin, Italy; ^2^Dipartimento di Scienze Medico-Veterinarie, Università di Parma, Parma, Italy; ^3^Allevamento Le Fontanette, Vigone, Italy

**Keywords:** distal intermediate ridge of the tibia, medial malleolus, lateral trochlear ridge, osteochondrosis, tarsocrural joint, C-terminal cross-linked telopeptides of type II collagen, leukotriene B4, prostaglandin E2

## Abstract

Tarsocrural osteochondrosis (OCD) is a developmental orthopedic disease commonly affecting young Standardbreds, with different fragment localization and size. Clinically, it is characterized by variable synovial effusion in the absence of lameness, whose determinants are ill-defined. We hypothesized that localization and physical characteristics of the osteochondral fragments like dimensions, multifragmentation, and instability influence joint effusion and correlate with synovial markers of cartilage degradation and inflammation. Clinical data, synovial fluid and intact osteochondral fragments were collected from 79 Standardbred horses, aged between 12 and 18 months, operated for tarsocrural OCD. The severity of tarsocrural joint effusion was assessed semi-quantitatively. The osteochondral fragment site was defined radiographically at the distal intermediate ridge of the tibia (DIRT), medial malleolus (MM) of the tibia, and/or lateral trochlear ridge (LTR) of the talus. Size, stability, and arthroscopic appearance (unique or multi-fragmented aspect) of the fragments were determined intra-operatively. Synovial concentrations of C-terminal cross-linked telopeptides of type II collagen (CTX-II), leukotriene B4 (LTB4), and prostaglandin E2 (PGE2) were quantified. Tarsocrural synovial effusion was significantly affected by localization and stability of the fragments, with MM-located and unstable fragments being associated with highest joint effusion. Concentrations of CTX-II, LTB4, and PGE2 positively correlated with the severity of synovial effusion. This study underlines characteristics of the osteochondral fragments determining higher synovial effusion in OCD-affected tarsocrural joints and suggests both inflammation and extra-cellular matrix degradation are active processes in OCD pathology.

## Introduction

Osteochondrosis (OCD) is a developmental orthopedic disease of the equine diarthrodial joints characterized by an initial ischemic injury in the growing epiphyseal cartilage, which may later result in partial detachment of an osteochondral fragment from the line between the retained hyaline cartilage and the normal bone, in turn leading to deterioration of the overlying articular cartilage (1, 2). Complete detachment of the osteochondral fragments results in loose bodies floating in the affected synovial cavity. Causes of the initial ischemic injury are incompletely understood, and limited evidence exists in support of hypotheses that have been postulated on this regard. Insufficient arterial branching (3), weakness of the collagen matrix in the cartilage leading to insufficient mechanical support to the vascularization bed (4), and mechanical shearing of cartilage canals at some predilection sites (5) could all contribute to a detrimental reduction in blood supply to a rapidly growing epiphysis.

The tarsocrural joint is the most commonly OCD-affected joint in Standardbred (STBs). In Norwegian bloodlines, tarsocrural OCD has 19.3% reported prevalence (6) and its heritability is estimated to be 0.52 (7). Tarsocrural OCD lesions may localize to the distal intermediate ridge of the tibia (DIRT), medial malleolus (MM), and lateral trochlear ridge (LTR) of the talus (6, 8). OCD in the DIRT showed no evolution between the ages of 6 and 18 months based on a study in a large cohort of young horses (9), confirming the absence of radiological change after 7–8 months of age (9). Clinically, the severity of tarsocrural joint effusion associated with OCD can be highly variable, but rarely it is accompanied by lameness (5, 10). Early intervention for fragment removal is currently the preferred option for OCD treatment, in both symptomatic and asymptomatic cases, guided by two retrospective clinical studies (11, 12). However, fragment removal *per se* is not resolutive in all cases, as operated horses were still shown to have reduced race starts when OCD was bilateral and LTR was affected (12).

The available evidence supports a role for synovial inflammation in tarsocrural OCD (10, 13), associated with hyaluronic acid degradation (14). Evidence also exists supporting a role for cartilage degradation as a further mechanism implicated in equine OCD (15, 16). Given the pro-inflammatory properties of some cleavage product of type II collagen, such Coll2-1 (17), further efforts are warranted to clarifying the mutual relationship between these factors as well as their contribution to the clinical presentation of the disease.

This study was performed on a cohort of STBs undergoing early arthroscopic surgery with the aim to explore the influence of tarsocrural OCD lesion site and physical characteristics of the fragments on the severity of joint effusion, synovial fluid inflammation and cartilage degradation biomarkers. Also, the mutual relationship between the latter three variables was explored. Our hypotheses in the current work were that joint effusion is positively associated with OCD fragments localized at the MM, and with large, multi-fragmented, and unstable fragments, as well as with increasing concentration of inflammation and cartilage remodeling biomarkers. As a relevant addendum to this work, we describe the histological characteristics of the collected fragments.

## Materials and methods

A retrospective cross-sectional study was conducted using data and samples collected for other research purposes at our institution starting from 2012. Clinical details, radiographical data, synovial fluid, surgical records, and OCD lesion samples of 12–18-month-old STB yearlings with tarsocrural OCD referred to our Veterinary Teaching Hospital (VTH) for arthroscopic surgery (cases) were studied. Data on OCD lesion site, size, stability, and arthroscopic appearance were retrospectively assessed and compared to joint effusion. Synovial fluid concentration of inflammatory and structural biomarkers was also assessed in OCD-affected joints and compared with samples obtained from tarsocrural synovial fluid of age-matched yearlings euthanized at the VTH for unrelated clinical reasons and used as controls. Lastly, histology was performed on OCD fragments removed arthroscopically without macroscopic damage.

### Study population

The database of the clinical management software of our VTH, namely Provet Cloud®, was interrogated, and clinical records retrospectively assessed. “Osteochondrosis,” or “Standardbred,” or “hock,” or “tarsocrural joint” were the keywords used for case search. Cases were included in this study if they met the following criteria: (1) being referred for tarsocrural OCD fragment removal from the 1st of January 2012 through the 31st of December 2022; (2) being 12–18-month-old at surgery; and (3) having synovial fluid samples obtained at surgery still available. Data acquired from the digital clinical records were age and sex of the horse, hock radiography, date of the surgery, surgery report and recording, days of hospitalization, and post-operative complications. All cases were operated by the same surgeon. Data were collected with the owners’ consent, and the research was conducted with the approval of our institution’s Commission for Ethics and Animal Welfare. Horses included in the study were from a limited number of farms in the same geographical area. Yearlings were managed living free in large paddock within groups of animals of the same gender.

### Control group

Ten age-matched horses, euthanized for reasons unrelated to this study at our VTH, between January 1st and July 30th, 2023, were included as controls. Six out of 10 controls are STBs, two out of 10 are Quarter Horses and 2 out of 10 are Warmbloods. Five out of 10 yearlings were euthanized for orthopedic problems unrelated to the hock joints: long bone fractures [1], stifle joint dysplasia [1], atlas fracture [1], fracture of the pelvis [1], and *Rhodococcus* osteomyelitis [1]. Other horses were euthanized after a diagnosis of cervical ataxia [2], colic without surgical option [1], atypical myopathy [1], and pleuropneumonia [1]. The age of the control group was 15 ± 2 months (mean ± SD; range 13–18-month-old). Horses were excluded if they had signs of tarsocrural joint abnormalities at necropsy. A synovial fluid sample was collected immediately after euthanasia from the dorsomedial pouch of the joint and samples were handled as described for OCD cases.

### Clinical assessment

Cases were not evaluated for lameness due to the young age of the animals. Tarsocrural joint effusion was subjectively scored using the semi-quantitative grading system proposed by Bergin et al. (18) and modified by Brink et al. (10), based on the profile of the medial aspect of the joints between the medial malleolus and the distal tubercle of the talus, as follows: 0 = no joint effusion (concave profile on the medial aspect of the joint), 1 = mild joint effusion (slight bulging profile on the medial aspect of the joint), 2 = moderate joint effusion (clear bulging profile on the medial aspect of the joint), and 3 = severe joint effusion (obvious bulging profile in all the joint recesses). Radiographic examination of the tarsocrural joint were performed under sedation (detomidine 20 mcg/kg and buthorphanol 20 mcg/kg IV) with the following views: Dorso-Plantar (DP), Latero-Medial (LM), 45° Dorsolateral-Plantaromedial (DLPM) oblique and 45° Dorsomedial-Plantarolateral (DMPL) oblique (19). A further radiographic view (10° DLPM oblique view) was occasionally performed to better delineate fragmentations in the MM.

### Arthroscopic surgery

All surgeries (either uni- or bilateral) were performed at the VTH. Pre-operatively, single dose administration of penicillin (20,000 UI/kg IM), gentamycin (6.6 mg/kg IV), and phenylbutazone (4.4 mg/kg IV) occurred 30 min before induction of general anesthesia following the Swedish Veterinary Association’s Guidelines for the clinical use of antibiotic in horses (April 2013). Two arthroscopic ports were employed for the examination of the tarsocrural joint, with the optical port at the medial aspect of the joint, abaxially to the saphenous vein, and the operative port in the dorsolateral aspect of the joint (20). Horses with OCD lesions localized to the MM had two arthroscopic ports at the dorsomedial aspect of the joint, with the optical port axial, and the operative port abaxial to the saphenous vein (20). The tarsocrural joint was kept flexed during the procedure (approximately at a 90° angle), and the intraoperative pressure in the joint was set at 65–70 mm Hg by means of an arthroscopic pump. The OCD fragments were identified and removed using Ferris-Smith rongeurs of different size, based on fragment dimension. Fragments were identified macroscopically and by palpation with an arthroscopic probe, and removed either directly, after gently twisting the OCD fragment, or using sharp instruments to lift the fragments before removal. In the latter case, the instruments were advanced along the line of separation of the fragments with the parent bone. Care was taken not to break the fragment during removal. After fragment removal, the OCD site was debrided using spoon curettes until trabecular bone was observed. Joint lavage was continued with sterile saline solution during the procedure using an egress cannula, maintaining a high-flow fluid rate. Arthroscopic ports were closed using a single interrupted horizontal mattress suture (USP 0 or 2–0 polypropylene). When necessary, more sutures were employed to close the lateral port after removal of large fragments. All the operated joints were bandaged with a sterile light bandage at the end of the procedure and a sterile three-layer 8-figure bandage was then performed when the horse was standing in the recovery box. The arthroscopic portals were bandage-protected for 7–8 days after surgery. The first layer of the bandage was a cotton roll, followed by a cohesive bandage and an elastic third layer. This third layer was made by a custom-made elastic mesh with a zipper closure, to avoid compression on the calcaneal tendon.

Digital video recordings of the surgical procedures were routinely saved.

### Surgical report and records of assessment

The following information were obtained either by reviewing the surgical report and the digital recording:

1. Fragment site was classified as DIRT, MM, and/or LTR of the talus based on its anatomical location.
2. Fragment size was measured at the major axis diameter using a caliper after surgical removal. Fragments were classified as large, when their length was >12 mm for DIRT fragments, 8 mm for MM fragments, and 9 mm for LTR fragments or small, when their length was ≤ to the reported values. The cut-off values correspond to the mean size (length) of OCD fragments reported in a previous study on a population of STBs (21).
3. Arthroscopic appearance: unique ovoid fragment or multi-fragmented (cobblestone aspect).
4. Fragment mobility, based on a modification of the Guhl classification, developed for human arthroscopy. Guhl 1 lesions were defined as intact OCD lesions with a clear separation from the subchondral bone but highly stable when palpated with a probe. Guhl 2 lesions were defined as partially detached or loose bodies, easily movable from the subchondral bone using a needle or an arthroscopic probe (22).
5. Hemorrhage from the bed-site: this was evaluated at the time of fragment removal prior to debridement of the bed and classified as present, when bleeding required an additional fluid lavage to maintain adequate visibility during surgery, or absent.
6. Synovitis was classified using a simplified semi-quantitative score based on the arthroscopic scoring evaluation described in the work of Gangl et al. (23): mild (=1), moderate (=2), or severe (=3), based on the extension, redness, and severity of synovial membrane proliferation in the examined joints.

The presence of further lesions in regions of articular cartilage non affected by OCD was also noticed and recorded.

### Synovial fluid biomarkers

Synovial fluid samples were obtained by arthrocentesis of the operated joints through the dorsomedial recess of the joint at the beginning of the surgical procedure, using a 19-gauge 4-cm needle, with the saphenous vein and the medial malleolus as landmarks for the joint puncture. Approximately 6–8 mL of synovial fluid were withdrawn in EDTA-containing tubes and placed on ice. Within 1 h from collection, the samples were centrifuged at 1,600 RPM for 20 min at 4°C to remove cells and debris, and mixed with 1 mM phenylmethylsulphonylfluoride (PMSF, an inhibitor of serine protease). Synovial fluid aliquots (5 × 1 mL) were collected in plain tubes and stored at −80°C for subsequent analysis. After thawing and before being processed for ELISA tests, synovial fluids were treated with hyaluronidase (Hyaluronidase from bovine testes, Sigma H3884, Sigma-Aldrich, Saint Louis, MO, USA) at a concentration of 20 UI/mL for 30 min at 37°C to reduce viscosity and diluted 1:2 with HPE-0.1375% Tween buffer solution (Sanquin Reagents, Amsterdam, Netherlands). Synovial concentrations of C-Terminal cross-linked telopeptides of type II collagen (CTX-II), leukotriene B4 (LTB4), and prostaglandin E2 (PGE-2) were measured using ELISA tests, previously validated for use in horses (24–26). For CTX-II we employed a commercially available sandwich ELISA kit (code AC-08 F1, Serum Pre-Clinical Cartilaps, IDS), for LTB4 and PGE-2 we employed commercial competitive ELISA kits for the quantitative determination of leukotriene B4 and Prostaglandin E2 in biological fluids (Leukotiene B4 Multispecies competitive Elisa Kit Thermo Fisher Scientific, Prostaglandin E2 Human Competitive Elisa Kit Thermo Fisher Scientific). Absorbance was measured at 450 nm with a microplate reader. Each sample was analyzed in duplicate. Positive, negative, and blank controls were included on each plate in duplicate. Intra- and inter-assay coefficients of variability (%CV) were calculated for each series of assays.

### Histology

Only those osteochondral fragments extracted intact during arthroscopic procedures were further processed for histology. Immediately after removal, they were placed in 10% buffered formalin for a minimum of 72 h and then decalcified in EDTA Disodium Salt acid buffer (Bio-Optica^®^ Osteodec 60-00-4) solution until low-voltage radiographic examination of the specimens confirmed that decalcification was completed. After removal from the decalcifying solution, fragments were rinsed under running water, cut in two halves along their maximal diameter and perpendicularly to their basal side (facing the trabecular bone), dehydrated, and paraffin embedded. Five-micron sections were mounted on slides and stained with hematoxylin and eosin (HE), and Masson’s trichrome stains. Specimens were evaluated by an experienced pathologist (EB) using a semiquantitative score designed for this study and based on the histological evaluation of the OCD fragment in the humane knee (27). The following separate histological sub-items were considered: ‘amount of fibrous tissue at the base of the fragment,’ ‘neovascularization,’ ‘necrotic bone trabeculae,’ ‘necrotic fibrocartilaginous tissue,’ ‘bone resorption,’ and ‘degenerated articular cartilage.’ All the sub-items were graded using a 0–2 scale where: ‘0’ is none, ‘1’ is small amount, and ‘2’ is large amount.

### Statistical analysis

Analyses were conducted using Prism v10.0 (Graphpad Software Inc., USA) and STATA v18 software (StataCorp LLC, USA). Data distribution for continuous variables was assessed by visual inspection of histograms and QQ-plots and by means of Kolmogorov–Smirnov statistical testing. Data are expressed as proportions for binomial variables, as median and interquartile ranges for non-parametric variables, and as mean ± standard deviation (SD) for parametric variables. The relationship between synovial effusion and its potential determinants (horse age at surgery, OCD lesion site, size of the fragment, mobility, and fragmentation pattern) was assessed with the multilevel mixed effect ordered logit model, also known as the proportional odds model, using robust cluster error, where the horse was treated as the cluster variable. Synovial effusion scores 0 and 1 were unified due to the low number of joints scoring 0 (*n* = 3). The relationship between hemorrhage after fragment removal and potential determinants (age at surgery, OCD lesion site, size, mobility, and fragmentation pattern) was assessed with a logit model, using robust cluster error, where the horse was treated as the cluster variable.

Fragments with cobblestone aspect were considered multi-fragmented OCD in the statistical analysis. Tarsocrural joints with multiple OCD sites were included in the logit model and the logistic model adjusted for the clustering effect. Logarithmic transformation was employed for statistical analyses of biomarker concentration data. Differences in biomarker synovial concentrations were tested using t-test. Correlations between biomarker synovial concentrations and synovial effusion were tested using Spearman correlation. The effect of OCD lesion site, fragment’s dimension, osteochondral fragmentation, and mobility score (Guhl 1 vs. Guhl 2) on biomarkers synovial concentrations was studied by means of two-way ANOVA, with Bonferroni correction for post-hoc tests. The variable “age” of the animals was included in this statistical analysis as a covariate.

To explore the relationship between histological findings and OCD lesion stability, logistic model was employed in which histological findings of DIRT (*n* = 27) and LTR (*n* = 1) lesions were grouped and used as controls compared to those of MM (*n* = 8). Bivariate logistic models were initially employed to assess the effect of different histological sub-items singularly on the logistic model. Alpha was set at 0.05 for multivariate analyses.

## Results

### OCD cases

Seventy-nine yearlings were included in the study, 42 males and 37 females, accounting for 118 tarsocrural joints and 124 OCD lesions. All horses were referred from five farms located in the geographical area of the metropolitan city of Turin, participating in a pre-sale radiographic screening program run by our institution. The horses studied underwent surgery in a narrow range of age (16 ± 1 month is the mean ± SD; range: 12–18 months) and were hospitalized for 3 ± 1 days (range 2–7 days). Complications were observed in 2/118 operated joints (2/79 surgical sessions), namely, the development of a post-operative septic synovitis and a surgical site infection localized to the lateral arthroscopic portal. These two cases were hospitalized for 65 and 8 days and were not included in the calculation of the mean hospitalization time.

### Clinical and arthroscopic findings

**Figure 1:**
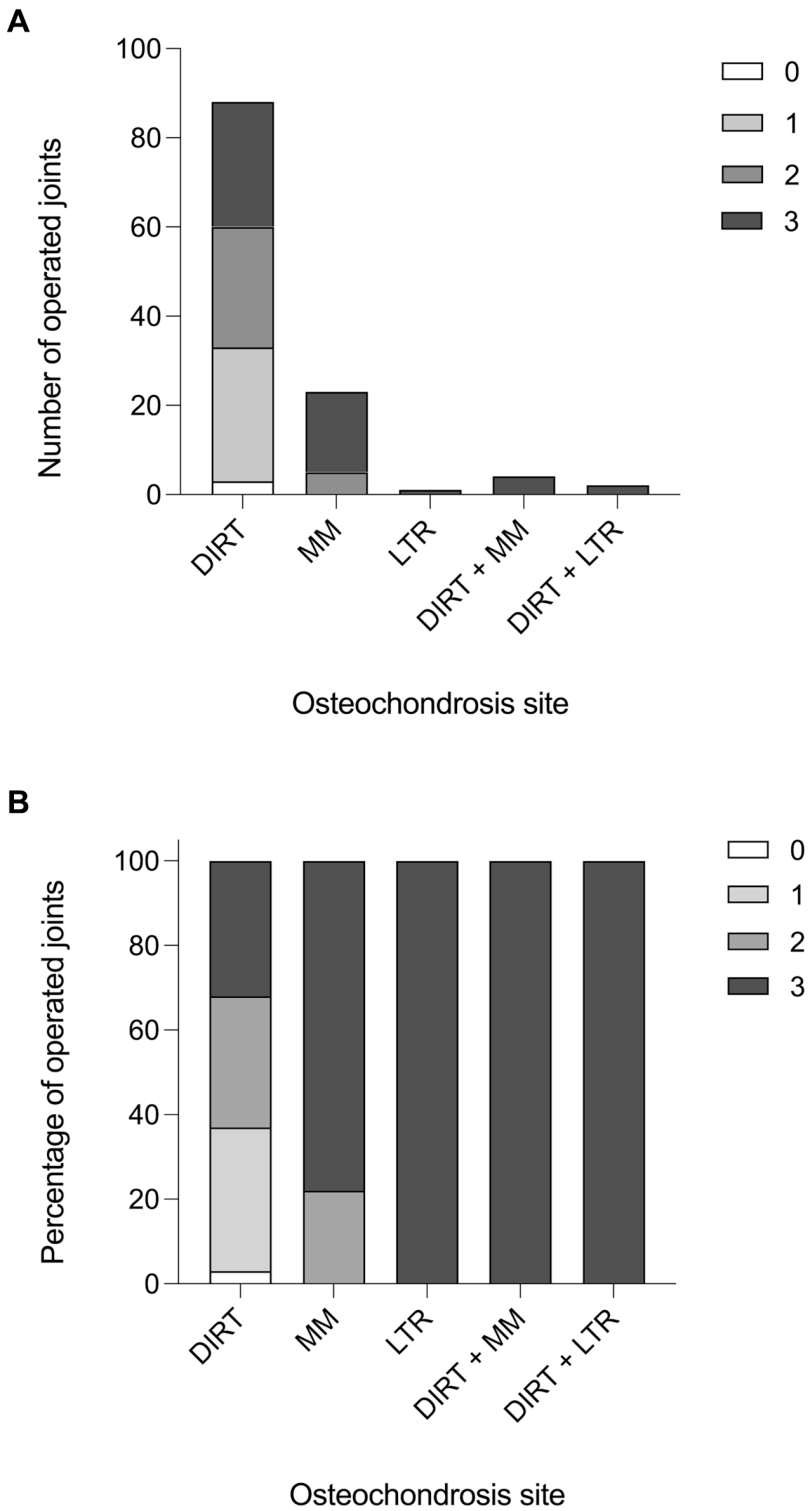
Synovial effusion score. **(A)** Histogram reporting the effusion score in the operated tarsocrural joints in relation to the localization of the fragment (number of joints). **(B)** Histogram reporting the effusion score in the operated tarsocrural joints in relation to the localization of the fragment (in percentage).

Forty-two out of 79 (53%) horses studied had a single tarsocrural joint affected, whilst 37 horses (47%) had both tarsocrural joints affected. Among the 42 cases with unilateral OCD lesions, 26 (62%) right hindlimbs and 16 (38%) left hindlimbs were affected. Three out of 118 (2.5%) tarsocrural joint scored 0 for synovial effusion, 30 (25.4%) scored 1, 32 (27.1%) scored 2, and 59 (50.0%) scored 3. Representative histograms of the synovial effusion score are presented in Figure 1.

In the population studied, 94 out of a total of 124 OCD lesions were localized at DIRT (75.8%), 27 at MM (21.8%), and 3 at LTR of the talus (2.4%) (Table 1). Radiographically, OCD lesions were detected at a single site in 112 over 118 (95%) joints, and at two sites in six joints (5%; six horses). In the latter case, DIRT and LTR sites were affected in two joints, whilst DIRT and MM sites were affected in the remaining four joints.

**Table 1:** Physical characteristics of the fragments and related classification taken in account in our study.

	*N*	Hemorrhage		Size		Fragment		Mobility			
		Yes	No	Large	Small	Unique ovoid	Multi-fragmented	Guhl 1	Guhl 2	OCD lesion size (mean ± SD)	Synovitis median (range)
DIRT	94	23 (24.5%)	71 (75.5%)	53 (56.3%)	41 (43.7%)	64 (68.1%)	30 (31.9%)	43 (45.7%)	51 (54.3%)	11.5 ± 4.5	1 (1; 2)
MM	27	17 (63.0%)	10 (37%)	8 (29.6%)	19 (70.4%)	24 (88.9%)	3 (11.1%)	22 (81.5%)	5 (18.5%)	9.3 ± 3.0	2 (2; 2)
LTR	3	1 (33.3%)	2 (66.7%)	2 (66.6%)	1 (33.4%)	3 (100%)	0 (0%)	1 (33.3%)	2 (66.7%)	17.3 ± 14.7	1 (1; 2)
Total	124	41 (33.1%)	83 (66.9%)	63 (50.8%)	61 (49.2%)	91 (73.4%)	33 (26.6%)	66 (53.2%)	58 (46.8%)	11.2 ± 4.7	2 (1; 3)

Considering the joint factors only the synovitis score was reported.

**Figure 2:**
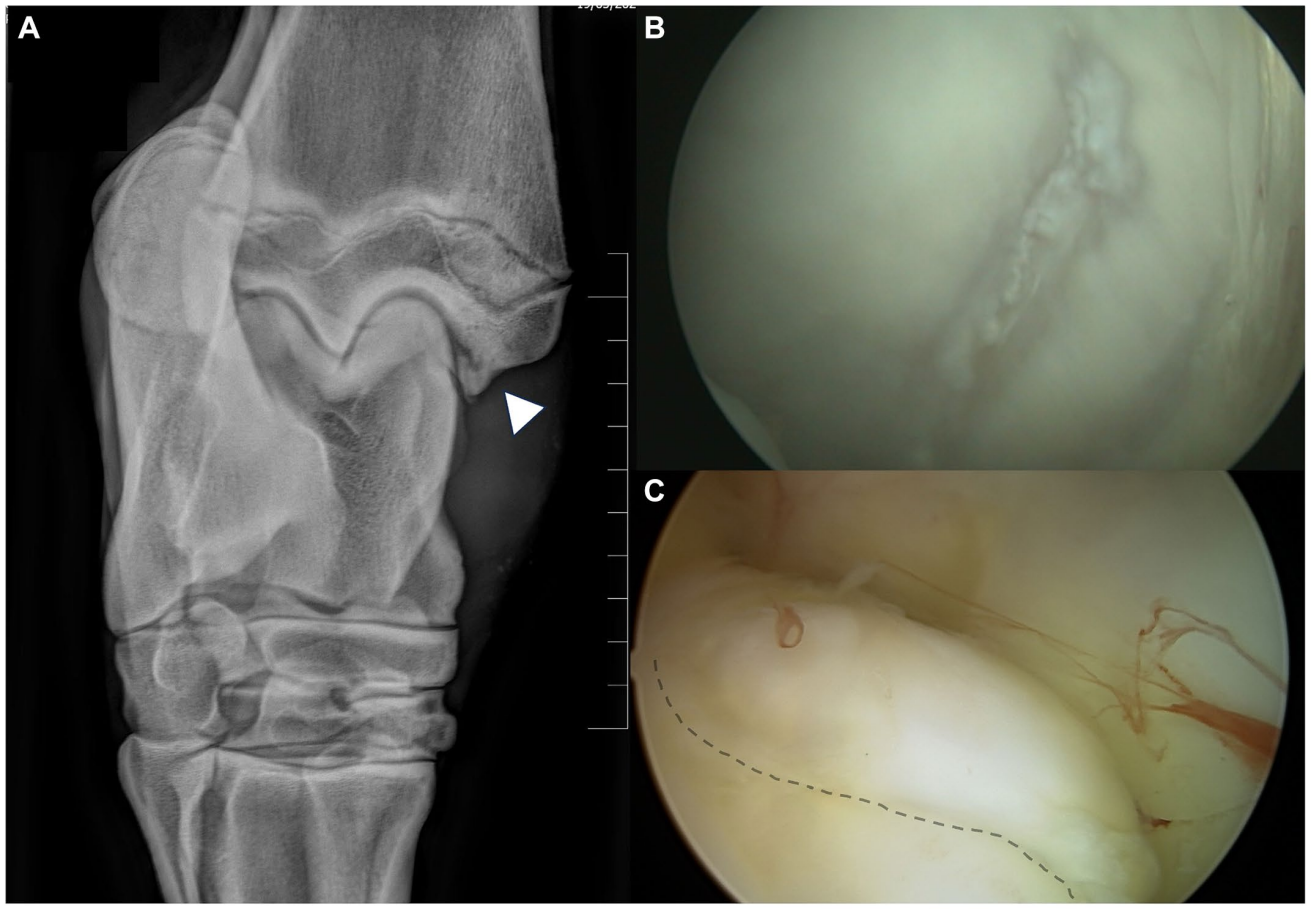
Regions of chondromalacia observed in case #107, associated with MM-OCD. **(A)** The 10°–20° DorsoLateral-PlantaroMedial Oblique radiographic view of the tarsocrural joint is the best to highlight the medial malleolus fragments. The fragment (arrowhead) is clearly visible in the axial aspect of the medial malleolus. **(B)** The abaxial aspect of the medial trochlear ridge (MTR) of the talus in this case with MM-OCD shows an elongated area of chondromalacia in the region that interfere with the OCD lesion at maximal flexion of the joint. **(C)** The MM is visualized with the arthroscopic port localized in the medial pouch of the tarsocrural joint. The elongated OCD fragment is detectable at the axial aspect of the malleolus (dotted line).

During arthroscopy recording review, it was determined that 19 operated joints (15.3%) had mild, 38 (30.6%) had moderate, and 67 (54.1%) had severe synovitis. Most of the OCD lesions (*n* = 91, 73.3%) appeared like a single and unique fragment, while 33 (26.7%) showed a multi-fragmentary aspect. As for mobility, 66 lesions (53.2%) were classified as Guhl 1 and 58 (46.8%) as Guhl 2 (Table 1 and Supplementary Figure 1). Regions of chondromalacia were observed at the axial aspect of the MTR of the talus in nine horses with lesions at the MM, located exactly in the region facing the OCD at the maximal joint flexion (Figure 2). Hemorrhage from the fragment bedside was observed after arthroscopic removal of 41 OCD lesions (33%). Removed OCD fragments measured along the major axis diameter an average of 11.2 ± 4.7 mm (mean ± SD; range 6–34 mm). Based on the proposed classification, 61 lesions (49%) were classified as small and 63 (51%) as large (Table 1).

### Relationship between synovial effusion and clinical and arthroscopic findings

The OCD lesion site and mobility score were significantly associated with synovial effusion in the population studied (Table 2). In detail, the multivariate hierarchical model employed showed that MM- and LTR-OCD were associated with increased synovial effusion compared to DIRT-OCD, and Guhl 2 OCD lesions were associated with increased synovial effusion compared to Guhl 1 lesions. In terms of probability, joints with OCD localized at MM and LTR had increased probability to display a severe (grade 3) synovial effusion compared to DIRT. Also, Guhl 2 lesions had higher probability to display a severe (grade 3) synovial effusion compared to Guhl 1 lesions (Table 2).

**Table 2:** Determinants of synovial effusion in the population studied (*N* = 124).

	*β* Coefficient (95% CI)	*p* value	Probability of effusion 0_1 (mean ± SD)	Probability of effusion 2 (mean ± SD)	Probability of effusion 3 (mean ± SD)
Age [months]	−0.099 (−0.476; 0.268)	0.60	–	–	–
OC site					
*Ref: DIRT*	–	–	0.36 ± 0.37	0.29 ± 0.14	0.35 ± 0.33
**MM**	**5.546 (2.568; 8.524)**	**0.000**	**0.01 ± 0.01**	**0.14 ± 0.11**	**0.85 ± 0.12**
**LTR**	**25.763 (23.833; 27.692)**	**0.000**	**0.00 ± 0.00**	**0.00 ± 0.00**	**1.00 ± 0.0**
Lesion size					
*Ref: small*	–	–	–	–	–
Large lesion	0.459 (−0.556; 1.474)	0.37	–	–	–
Multifragmented lesion					
*Ref: 1 fragment*	–	–	–	–	–
≥1 fragment	−0.754 (−1.952; 0.444)	0.22	–	–	–
Mobility					
*ref: Guhl 1*	–	–	0.49 0.37	0.21 ± 0.11	0.30 ± 0.39
**Guhl 2**	**4.767 (2.251; 7.283)**	**0.000**	**0.03 ± 0.03**	**0.29 ± 0.17**	**0.68 ± 0.19**

The bold values represent the statistically significant values.

### Relationship between hemorrhage from the OCD site and clinical and arthroscopic findings

The OCD lesion mobility score was the only variable significantly associated with the occurrence of hemorrhage from the parent bone after OCD lesion removal. Guhl 2 lesions had a reduced likelihood of being associated with hemorrhage at the time of surgical removal (Table 3). In other words, Guhl 2 fragments had 0.91 ± 0.04 probability of not being associated with hemorrhage, compared to 0.45 ± 0.13 of Guhl 1 lesions. Table 4 summarizes mean values per group.

**Table 3:** Determinants of hemorrhage after OC fragment detachment from the subchondral bone (*N* = 124).

	*β* Coefficient (95% CI)	OR (95% CI)	*p* value
Age [months]	−0.178 (−0.514; 0.158)	0.84 (0.60; 1.17)	0.300
OC site	–	–	–
*Ref: DIRT*	–	–	–
MM	1.211 (−0,082; 2.505)	3.358 (0.921; 12.242)	0.066
LTR	22.563 (20.710; 24.416)	2.180 (0.445; 10.660)	0.336
Lesion size	−0.206 (−1.168; 0.755)	0.813 (0.311; 2.128)	0.674
*Ref: small*	–	–	–
Multifragmented lesion	0.041 (−1.082; 1.164)	1.042 (0.339; 3.203)	0.943
*Ref: 1 fragment*	–	–	–
**Mobility**	**−2.193 (−3.336; −1.050)**	0.111 (0.035; 0.350)	**0.000**
*Ref: Guhl 1*	–	–	–

The bold values represent the statistically significant values.

**Table 4:** Hemorrhage frequency classified by OC lesion site and mobility score (*N* = 124).

		DIRT		MM		LRT	
		Guhl 1	Guhl 2	Guhl 1	Guhl 2	Guhl 1	Guhl 2
Hemorrhage +		20	3	15	2	1	0
Hemorrhage −		23	48	7	3	0	2
	Subtotal	43	51	22	5	1	2
	Total	94		27		3	

### Synovial fluid biomarkers and their relationship with clinical and arthroscopic findings

**Figure 3:**
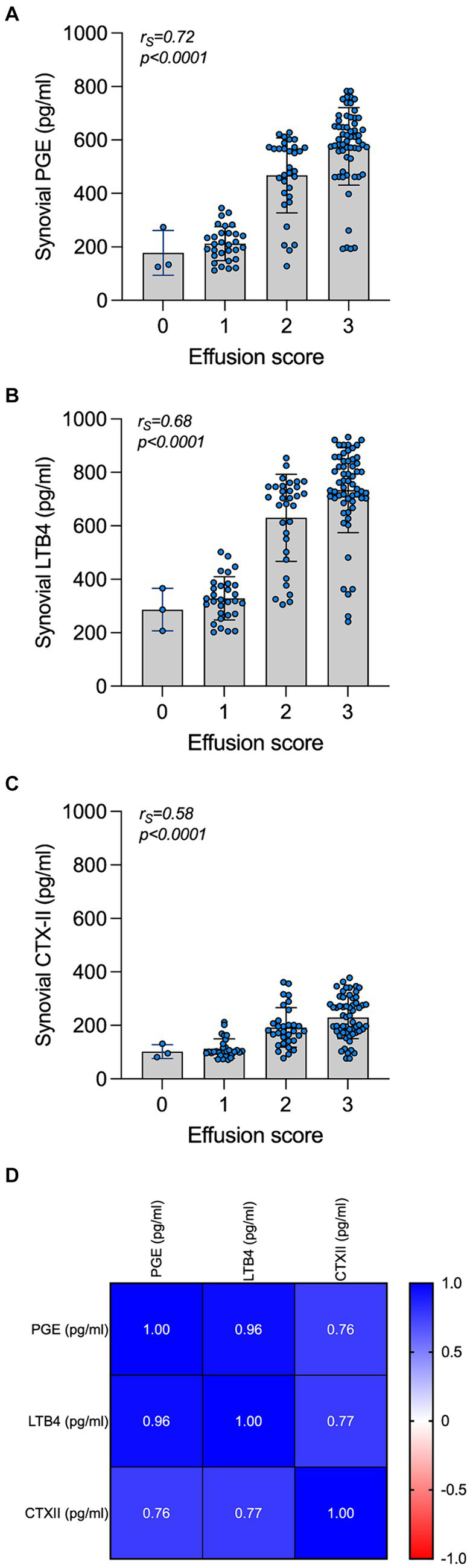
Synovial concentration of PGE, LTB4, and CTX-II and correlation with the joint effusion score and with each other. Synovial concentration of PGE **(A)**, LTB4 **(B)**, and CTX-II **(C)** in OCD cases are grouped by the subjective clinical joint effusion score. The correlation of the biomarkers and the joint effusion are listed at the top of each graph. A correlation matrix reporting values of correlations in the synovial fluid of cases is presented at the bottom **(D)**.

Biomarkers were always within the minimal detection values of the immunoassays. An intra-assay %CV < 10% and an inter-assay %CV < 15% were considered acceptable. The synovial concentrations of PGE, LTB4, and CTX-II were significantly increased in OCD compared to control joints (*p* < 0.0001 Welch’s t test; Table 5). The effect of the covariate “age” was not significant on the concentration of biomarkers examined in this study. In OCD-affected joints, the synovial concentrations of PGE, LTB4, and CTX-II significantly and positively correlated with each other as well as with the joint effusion score (Figure 3). The concentration of PGE, LTB4, and CTX-II was significantly affected by lesion site and mobility (*p* < 0.001 for all). Also, a significant interaction between these two variables was observed (*p* < 0.05). In detail, PGE, LTB4, and CTX-II were significantly higher in cases with Guhl 2 OCD localized at the DIRT and LTR, compared to cases with Guhl 1 OCD. Guhl 1 lesions in the MM had significantly higher values of PGE, LTB4, and CTX-II compared to Guhl 1 lesions at DIRT and LTR (Table 5).

**Table 5:** Synovial concentration of PGE, LTB4, and CTX-II in OCD cases (*N* = 118) and controls (*N* = 10).

	*N*			PGE [pg/mL]			LTB4 [pg/mL]			CTX-II [pg/mL]		
	Guhl 1	Guhl 2	Total	Guhl 1 (mean ± SD)	Guhl 2 (mean ± SD)	Total (mean ± SD)	Guhl 1 (mean ± SD)	Guhl 2 (mean ± SD)	Total (mean ± SD)	Guhl 1 (mean ± SD)	Guhl 2 (mean ± SD)	Total (mean ± SD)
Controls	–	–	10	–	–	86 ± 47	–	–	110 ± 86	–	–	26 ± 18
OCD	66	58	124	336 ± 193	581 ± 99	451 ± 198°	464 ± 216	751 ± 94	598 ± 222°	145 ± 67	239 ± 72	189 ± 84°
DIRT	43	51	94	229 ± 110	569 ± 96*	413 ± 198	344 ± 118	740 ± 91*	559 ± 224	115 ± 36	233 ± 72*	179 ± 83
MM	22	5	27	551 ± 135^#^	663 ± 70	572 ± 132	702 ± 165^#^	806 ± 78	722 ± 157	205 ± 73^#^	275 ± 72	218 ± 77
LTR	1	2	3	193	697 ± 121*	529 ± 304	363	880 ± 32*	708 ± 300	76	290 ± 21*	219 ± 124

*Significantly different from Guhl 1 within the same OCD lesion site group.

^#^Significantly different from DIRT and LTR within the same Guhl score group.

°Significantly different from the controls group.

### Histologic findings

**Figure 4:**
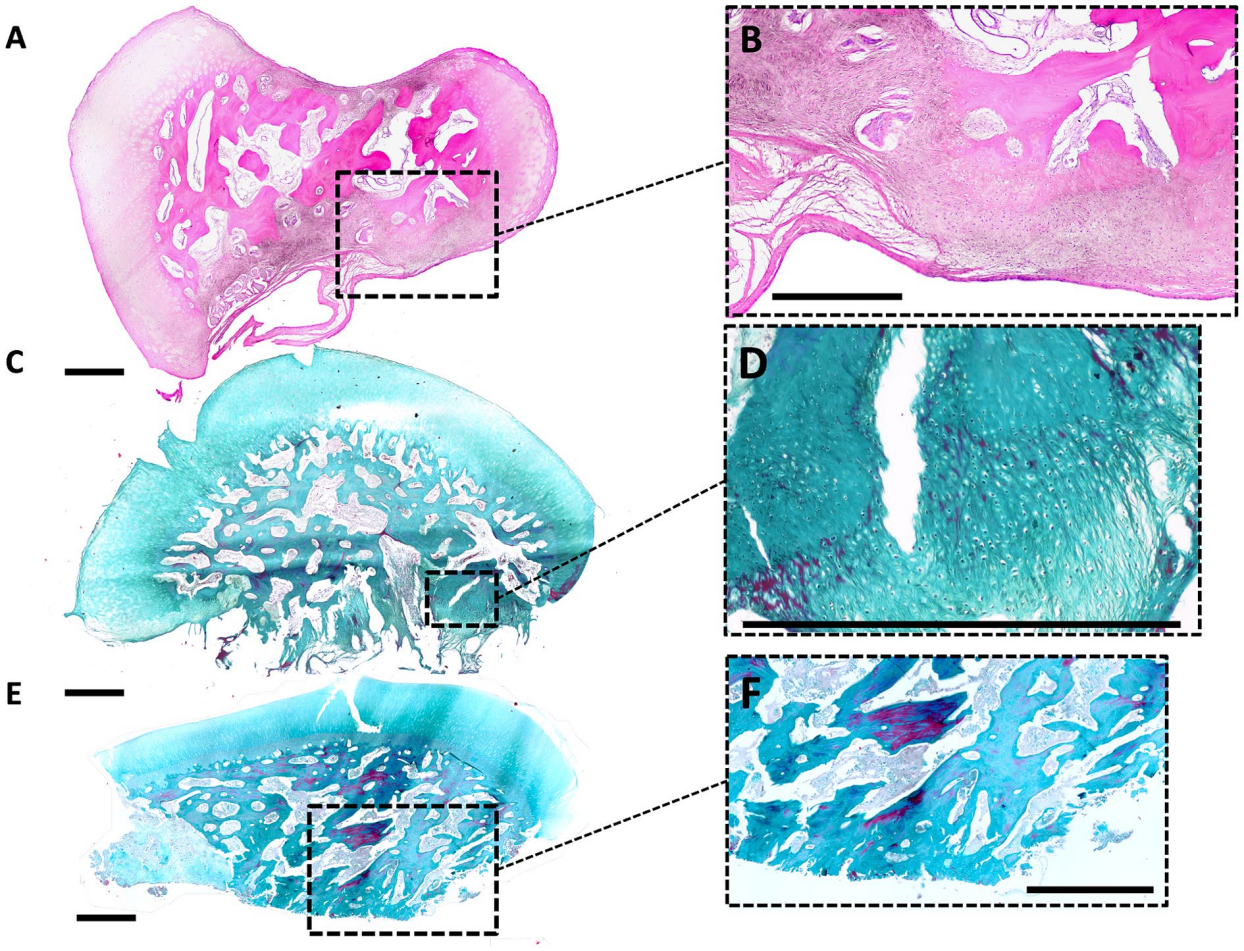
Histological findings in OCD fragments. **(A,B)** Histological image of a small and unstable DIRT-OCD fragment (small magnification, HE stain). The basal side, lower in the picture, shows a fibrous nonunion tissue which causes an unstable connection to the parent bone. In the opposite concave side, upper in the picture, is evident an indentation. Hyaline cartilage of the fragment is normal at the periphery, while the bone is characterized by a scarce number of trabecular connections. Degenerated islands of osteochondral tissue are present at the base of the fragment [Dotted square magnified in panel **(B)**]. **(C,D)** Large and unstable DIRT-OCD fragment (small magnification, Masson Trichrome stain). The basal side, lower in the picture, is characterized by the presence of fibrous nonunion tissue and fibrocartilage. This abnormal tissue is directly connected to the trabecular bone of the fragment [Dotted square magnified in panel **(D)**]. **(E,F)** Stable and small MM-OCD fragment (small magnification, Masson Trichrome stain). The shape of the fragment is more elongated than fragment in DIRT. The fragment is characterized by a well-demarcated connection line with the bone without fibrous tissue [Dotted square magnified in panel **(F)**]. The base of the fragment, lower in the picture, shows a large not remodeled area of osteochondral necrotic tissue (left side). The center of the fragment shows evident trabecular bone disconnection.

A total of 36 collected OCD lesions were processed for histology, of which 27 were from DIRT site, 8 from MM, and 1 from LTR. Fragments were all grossly ovoid in shape (Figure 4), presenting a basal side, facing the trabecular bone, and a synovial side, facing the joint. Complete decalcification was attained after three to 7 days in about half of the samples, while it took longer (up to 3 weeks) for the remaining samples.

Evaluation of the specimens using a semiquantitative histological score was reported in Table 6. The complete histological score data is available in the Supplementary material.

**Table 6:** Descriptive statistic of the histologic evaluation of the fragments.

Semiquantitative histological score	DIRT Fragments	MM Fragments	LTR Fragments
	Score 0–2	Score 0–2	Score 0–2
Amount of fibrous tissue at the base of the fragments	0 (1/27)	0 (7/8)	0 (0/1)
	1 (14/27)	1 (1/8)	1 (1/1)
	2 (12/27)	2 (0/8)	2 (0/1)
Neovascularization	0 (12/27)	0 (4/8)	0 (1/1)
	1 (11/27)	1 (4/8)	1 (0/1)
	2 (4/27)	2 (0/8)	2 (0/1)
Necrotic bone trabeculae	0 (7/27)	0 (0/8)	0 (0/1)
	1 (16/27)	1 (5/8)	1 (0/1)
	2 (4/27)	2 (3/8)	2 (1/1)
Necrotic fibrocartilaginous tissue	0 (18/27)	0 (3/8)	0 (0/1)
	1 (9/27)	1 (5/8)	1 (1/1)
	2 (0/27)	2 (0/8)	2 (0/1)
Bone resorption	0 (27/27)	0 (1/8)	0 (1/1)
	1 (0/27)	1 (6/8)	1 (0/1)
	2 (0/27)	2 (1/8)	2 (0/1)
Degenerated articular cartilage	0 (24/27)	0 (7/8)	0 (1/1)
	1 (3/27)	1 (1/8)	1 (0/1)
	2 (0/27)	2 (0/8)	2 (0/1)

The table summarizes the semiquantitative score evaluations on the separate histological sub-items.

The surface of the fragments shows hyaline cartilage with occasionally clusters of chondrocytes. Proceeding toward the basal side of the fragments, trabecular bone was encountered turning into an acellular dense fibrous tissue at the interface with the parent bone. Areas of neovascularisation were identified at the basal side of the fragments. Some MM lesions showed large areas of fibrovascular invasion into the center of the fragment, and an altered bone architecture. Degenerated islands of necrotic fibrocartilage were present in some fragments.

Results of the logit model reporting the relationship between histological variables, fragment’s position, and instability were detailed in Table 7. The probability of an unstable fragment is higher for each increase in fibrous tissue at the base of the fragment observed at the histological examination, while an increase probability of stable fragment is detected for each unit-increase in the neovascularization score.

**Table 7:** Logit model describing the relationship between single histological variables and fragment stability (ref: Guhl 2, unstable).

Unstable (Guhl 2)	Odds ratio	s.e.	*z* value	*p*-value	95% Conf. interval
Amount of fibrous tissue at the base of the fragments	18.6	19.66	2.76	**0.006**	2.33–147.91
Neovascularization	0.29	0.17	−2.03	**0.042**	0.093–0.95
Position of the fragment (MM)	0.043	0.051	−2.69	**0.007**	0.004–0.43
Necrotic fibrocartilaginous tissue	0.43	0.308	−1.17	0.24	0.11–1.73
Necrotic bone trabeculae	1.99	1.16	1.18	0.24	0.63–6.28
Bone resorption	1	–	2.28	0.022	1.14–6.01
Degenerated articular cartilage	0.68	0.73	−0.35	0.725	0.08–5.65

The bold values represent the statistically significant values.

## Discussion

The tarsocrural joint of STBs is commonly affected by OCD that persist until skeletal maturity of racehorses and can negatively impact their athletic career (3, 4, 6, 11). Our results provide evidence in support of OCD lesion site and instability as determinants of synovial effusion, in turn associated with increased pro-inflammatory mediators in the synovial fluid and extracellular matrix degradation. Lesions localized at MM and LTR of the talus were less frequently observed but, still, they were associated with higher score of joint effusion compared to DIRT localized lesions. Unstable lesions resulted in overall increased synovial effusion in the studied population. The DIRT lesions were unstable in approximately half of the cases. The LTR lesions were more commonly unstable than stable. The fragments from the MM are mostly classified stable. Interestingly, our raw data suggest an interaction might also exist between the site and the mobility score of the fragments. Unexpectedly, the size of the fragments was not significant associated with synovial effusion. Tarsocrural joints with multiple lesions (six joints in our study) presented all a severe synovial effusion score. In the joints with multiple fragments, we have a DIRT fragment associated to a MM or LTR fragment. Therefore, there are confounding factor (position and instability of the fragment) that are not independent from the effect of having multiple lesions in a joint in comparison to have a single OCD fragment. An age-correlation was not found significative with the joint’s effusion biomarker concentrations in the synovial fluid in our OCD group, however the horses examined in this study all fall within a very narrow age range.

The occurrence of hemorrhage during OCD lesion removal was associated with lesion stability, but this association was not significantly associated with the OCD site. Unstable lesions are easily movable using an arthroscopic probe, as they retained a loose fibrous attachment to the parent bone. These fragments were approximately 10 times less likely to result in hemorrhage from the parent bone during arthroscopic surgery compared to stable lesions, which instead required sharp instruments to break off their bony attachment. Animal studies showed that premature interruption of the epiphyseal cartilage blood vessels leads to necrosis of the epiphyseal cartilage at very young age (28). These necrotic areas result in failure of endochondral ossification, providing a region more vulnerable to repeated mechanical trauma (*osteochondritis manifesta*). In the LTR of the equine femur differences in the vascular tree of the femoral trochlear growth cartilage may trigger ischemic events at OCD-susceptible sites in foals (29). In the late-stage OCD, a vascular impairment of the lesion is well recognized. A vascular regression is observed in the late-stage fragments of pediatric knees subjected to MRI studies (30). It is reasonable to hypothesize that the hemorrhage score observed in surgery reflects the degree of vascularization of the OCD lesion (31). Unstable lesions or free-floating fragments would then have a reduced or absent vascular support (31), being completely dependent on the synovial fluid for their metabolism. Stable lesions could be more supported by vascularization from the underlying bone, retaining the ability for spontaneous healing (9). However, there is a lack of study on the role of vascularity in the late-stage OCD in animals. We know that OCD fragments refixation could result in reattachment of the lesion and we are aware that large flaps in the LTR of the equine stifle could spontaneously heal, meaning that a revascularization is possible, and it is essential for the OCD healing process. The degree of vascularity of the fragment from the underlying bone is going to be reduced for unstable late-stage fragments in the tarsocrural joints. A histological evaluation was performed in a limited number of fragments taken during surgery. Our results showed a disorganized deposition of collagen at the basal aspect of the fragments at the interface with the epiphyseal trabecular bone of unstable fragments. Such acellular fibrous tissue was interpreted as an attempt of reparative response, which however resulted in ineffective healing, like in non-union bone fracture (32). In other words, osteochondritis dissecans that persist over 12 months of age in tarsocrural joints are damaged osteochondral regions having an unsuccessful healing process. It seems reasonable to hypothesize that the amount of the fibrous tissue found at the interface between the OCD lesions and the underlying bone is strictly linked with the instability of the OCD and a reduced probability of hemorrhage during fragment removal. The MM lesions, featured by a very thin layer of collagen tissues at their basal side, were firmly attached to the bone and mostly scored stable. Contrarily, DIRT lesions, frequently classified as unstable, showed a thick layer of loose and disorganized collagen fibers, along their interface with the bone. Hemorrhage was higher in MM vs. DIRT lesions. MM lesions were often characterized by the presence of an area of bone remodeling and disconnected bone trabeculae, which could be linked to the activation of osteoclasts, that are brought by the vascular ingrowth into the fragment (31). We are not able to correlate the degree of hemorrhage with the neovascularization pattern observed at the histological examination due to the low number of examined fragments from this site.

The concentration of biomarkers in the synovial fluid provided a biological explanation for joint effusion in the OCD-affected joints. An increase concentration of PGE2, LTB4 and fibromodulin value were reported in the OCD-affected hock joints (13, 16). This is the first study showing an association between biomarkers and the score describing the severity of synovial effusion in the OCD-affected hock joints. An alteration in cartilage type-II procollagen and aggrecan content in synovial fluid was associated to OCD (33), and it was related with an excessive degradation of type-II collagen in the articular cartilage (34). It was demonstrated that bone fragments from OCD lesions stimulate *in vitro* the equine synovial lining cells to produce the inflammatory mediator PGE2 (35), providing a link between fragment degradation *in vivo* and its pro-inflammatory role. The trigger of synovial inflammation is not determined to date for any specific biomarker *in vivo*, but molecules derived by collagen degradation of late-stage OCD are candidate to be damage-associated molecular patterns. In the synovial environment, those molecules from fragment degradation can bind to their pattern-recognition receptors on or in synoviocytes, chondrocytes, and macrophages and initiate a pro-inflammatory feedback loop, via the activation of matrix-metalloproteases and cathepsin (36, 37). A further interesting finding that might have contributed to the elevated levels of biomarkers in presence of MM lesions is the association with cartilage erosion at the axial aspect of the MTR of the talus, even if MTR lesion only occurred in 9 of 27 MM cases in our study. The presence of devitalized bone might also explain why horses with OCD localized in the MM had significantly increased concentrations of pro-inflammatory mediators and cartilage degradation biomarker in the synovial fluid.

PGE2 controls in the current study are similar to the findings of De Grauw et al. (13), but LTB4 concentrations in controls joints for De Grauw et al. were more similar to the OCD samples in this study. We are not able to explain it except through difference in the population studied. Similarly, in the work of Cleary et al. (25), CTX-II concentrations in the fetlock and carpus of controls were between 100 and 200, and the same for yearlings in the study of Nicholson et al. (38). Again, this would be more like the OCD samples in the current study, not the controls. This is probably related to the different population sampled in our study.

Cross-linked C-telopeptide fragments of type II collagen (CTX-II) measures degradation of type II collagen via identification of a type II collagen C-telopeptide epitope (39). In horses, CTX-II concentrations have been shown to increase in animals with OA and are also affected by age. Synovial fluid concentrations of CTX-II in adult horses with osteochondral injury in the middle carpal and fetlock joints were significantly higher compared to radiographically normal joints (25). CTX-II values were increased in yearlings compared to adult horses and in osteochondral injured middle carpal joints compared to injured metacarpophalangeal joints (38). In STBs with post-traumatic osteoarthritis there was an increase in CTX-II concentrations over a 4-year period (40). Studies in horses confirmed the results of the research by Duclos et al. (41) conducted in a rabbit model of OA, where the anterior cruciate ligament were transected. In this study, the serum level of CTX-II correlated positively with histological score of the medial compartment of the knee in adult animals over 20 weeks. This is the first study where the CTX-II, a by-product of articular cartilage breakdown released by degenerating extracellular matrix was assessed in the synovial fluid of equine joints with OCD. The CTX-II was previously considered a specific biomarker of cartilage degradation (24) and osteochondral injury (38), but recently was found to primarily originate from the interface between subchondral bone and the articular cartilage or bone (42). In our study we are not able to define the source of this epitope in the synovial fluid in presence of OCD. Mechanical instability of the lesions could be the key point to explain the strong positive relationships observed between CTX-II, PGE2, and LTB4 levels within the synovial effusion. Our data support cartilage degradation products as a possible initiating cause of inflammation in late-stage equine OCD, but the opposite pathway is also a possible explanation of the kinetic of biomarkers observed in this study. We confirmed the link between OCD and an inflammatory pattern in the synovial fluid observed in previous studies (13, 16).

This study had several limitations. The first limitation of this case series is that the groups of horses with different OCD localizations are non-homogeneous in number, however this reflects the natural course of the disease in horses. We enrolled low number of control horses. Not all the controls were STBs, and this aspect may affect a different level of exercise for some horses. Furthermore, we included yearlings from farms in a specific geographical area; therefore, different population of STBs may have different results. For this study we analyzed the synovial fluid samples in two different clusters over time and with the second cluster we analyzed the controls as well. No effects related to the time of conservation were noted in our statistical model. However, we cannot exclude that data in the study are influenced by the length of storage time of the sample. A single degradative biomarker of the extracellular matrix was analyzed, and no direct measure of the expression of this biomarker was performed on the removed tissue.

## Conclusion

The current study shows for the first time that lesion site and instability can be considered two major determinants of joint effusion and related inflammation in equine tarsocrural OCD. Our data suggest that an abnormal reparative process of the osteochondral extracellular matrix is part of OCD pathogenesis, and its pathological manifestations may differ based on lesion site. Further work is warranted on this subject to explore more in depth the staging of lesions in relation to the specific site of equine OCD.

## Data availability statement

The original contributions presented in the study are included in the article/Supplementary material, further inquiries can be directed to the corresponding author.

## Ethics statement

The animal studies were approved by the Commissione di Etica e Benessere Animale. The studies were conducted in accordance with the local legislation and institutional requirements. Written informed consent was obtained from the owners for the participation of their animals in this study.

## Author contributions

AB: Conceptualization, Data curation, Formal analysis, Funding acquisition, Investigation, Methodology, Project administration, Resources, Software, Supervision, Validation, Visualization, Writing – original draft, Writing – review & editing. MP: Data curation, Investigation, Methodology, Supervision, Validation, Writing – original draft. EP: Data curation, Investigation, Methodology, Validation, Writing – original draft. DV: Formal analysis, Investigation, Methodology, Software, Writing – original draft. LB: Investigation, Methodology, Writing – review & editing. EB: Conceptualization, Data curation, Investigation, Methodology, Writing – original draft, Writing – review & editing. MB: Data curation, Formal analysis, Software, Supervision, Validation, Visualization, Writing – review & editing. BR: Conceptualization, Data curation, Investigation, Supervision, Visualization, Writing – review & editing.

## Supplementary material

Supplementary Figure 1 **(A)** Arthroscopic view of a multiple and large DIRT-OCD fragment, with the optical port inserted in the medial aspect of the tarsocrural joint and the operative port in the lateral pouch of the same joint. **(B)** Arthroscopic view of a small DIRT-OCD fragment using the same triangulation technique than in panel A. **(C)** Arthroscopic view of a stable DIRT-OCD fragment elevated from the parent bone using an atraumatic probe using the same triangulation than in panel A. **(D)** Arthroscopic removal of a round unstable DIRT-OCD fragment using a large size rongeur grasping forcep. The fragment is ready for histology after removal.
