# Supplementary figures and images for: Determinants of joint effusion in tarsocrural osteochondrosis of yearling Standardbred horses
Source: Front Vet Sci. 2024 Jul 24;11:1389798. doi: 10.3389/fvets.2024.1389798 (PMC11303144; doi:10.3389/fvets.2024.1389798)

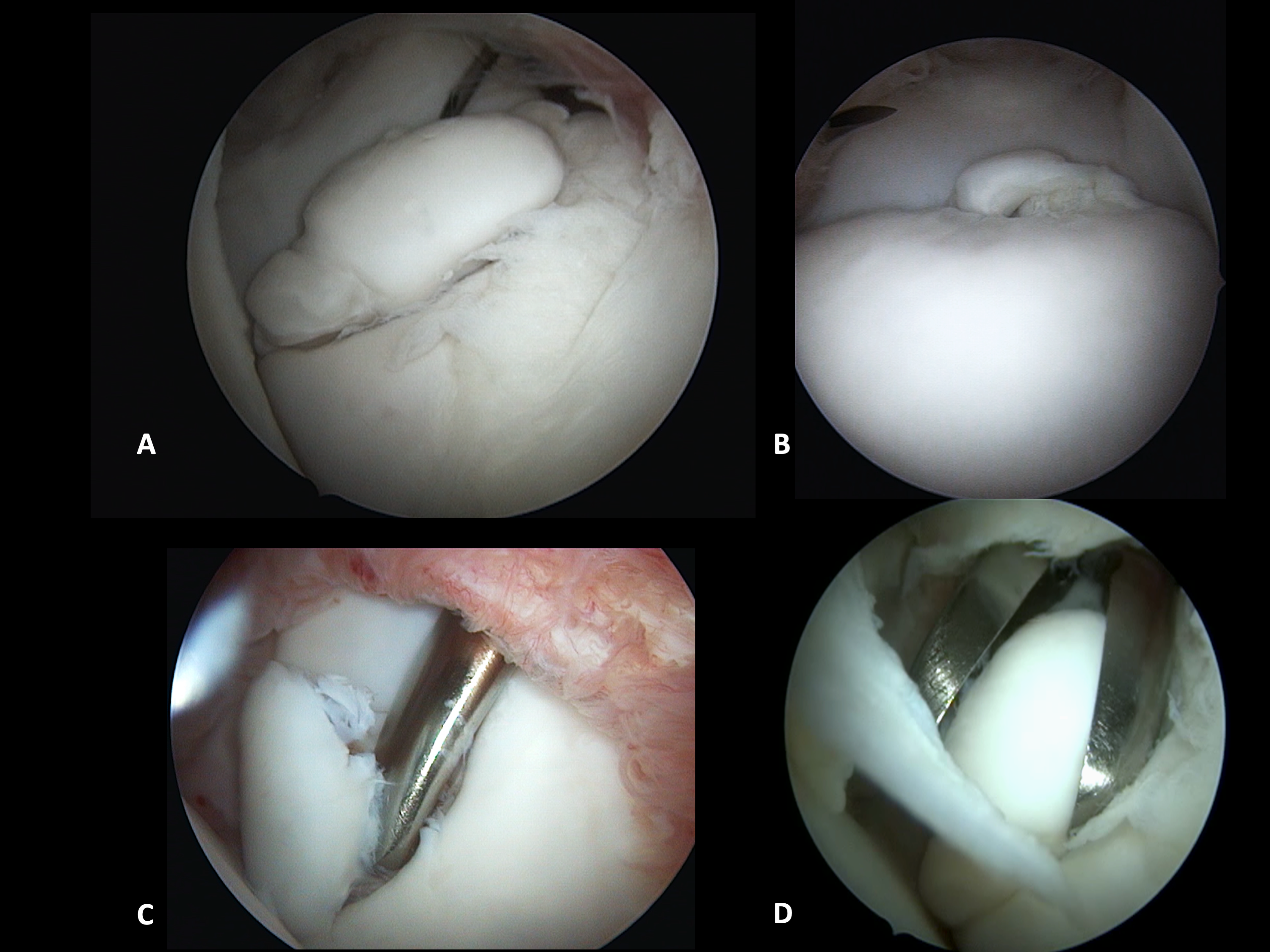

Supplement: Supplementary Figure 1 — (A) Arthroscopic view of a multiple and large DIRT-OCD fragment, with the optical port inserted in the medial aspect of the tarsocrural joint and the operative port in the lateral pouch of the same joint. (B) Arthroscopic view of a small DIRT-OCD fragment using the same triangulation technique than in panel A. (C) Arthroscopic view of a stable DIRT-OCD fragment elevated from the parent bone using an atraumatic probe using the same triangulation than in panel A. (D) Arthroscopic removal of a round unstable DIRT-OCD fragment using a large size rongeur grasping forcep. The fragment is ready for histology after removal. [file Image_1.TIFF]
