# Supplementary material for: Determinants of joint effusion in tarsocrural osteochondrosis of yearling Standardbred horses
Source: Front Vet Sci. 2024 Jul 24;11:1389798. doi: 10.3389/fvets.2024.1389798 (PMC11303144; doi:10.3389/fvets.2024.1389798)
Supplement: Supplementary file 2 [file Table_1.docx]

Supplementary Material

**Supplementary figure 1**

**Arthroscopic views of the operated osteochondral fragments**

A. Arthroscopic view of a multiple and large DIRT-OCD fragment. The optical port was inserted in the medial aspect of the tarsocrural joint and the operative instrumental port in the lateral pouch of the joint.

B. Arthroscopic view of a small DIRT-OCD fragment using the same triangulation technique described in panel A.

C. Arthroscopic view of a stable DIRT-OCD fragment elevated from the parent bone using an atraumatic probe.

D. Arthroscopic removal of a round unstable DIRT-OCD fragment using a large size rongeur grasping forceps. The fragment is ready for histology after removal.

**Supplementary Table**

**Histological findings in 36 osteochondral fragments**

| **ID Fragment** | **Position** | **Stability**  **(Guhl 1-2)** | **Fibrous tissue**  **(0-2)** | **Neovascularization**  **(0-2)** | **Necrotic fibrocartilaginous tissue**  **(0-2)** | **Necrotic trabeculae**  **(0-2)** | **Bone resorption (trabecular)**  **(0-2)** | **Degenerated articular cartilage**  **(0-2)** |
| --- | --- | --- | --- | --- | --- | --- | --- | --- |
| 91/23 | DIRT | 1 | 1 | 1 | 1 | 0 | 0 | 0 |
| 92/23 | DIRT | 2 | 2 | 0 | 0 | 1 | 0 | 1 |
| 93/23 | DIRT | 2 | 2 | 0 | 0 | 2 | 0 | 0 |
| 94/23 | DIRT | 2 | 2 | 0 | 0 | 1 | 0 | 0 |
| 95/23 | DIRT | 1 | 1 | 1 | 0 | 0 | 0 | 1 |
| 96/23 | DIRT | 2 | 1 | 0 | 0 | 1 | 0 | 0 |
| 97/23 | DIRT | 1 | 2 | 2 | 0 | 1 | 0 | 0 |
| 98/23 | DIRT | 2 | 1 | 1 | 1 | 2 | 0 | 0 |
| 99/23 | DIRT | 2 | 2 | 1 | 0 | 2 | 0 | 0 |
| 100/23 | DIRT | 1 | 1 | 2 | 0 | 1 | 0 | 0 |
| 101/23 | DIRT | 2 | 2 | 1 | 1 | 2 | 0 | 0 |
| 102/23 | MM | 1 | 1 | 1 | 1 | 2 | 1 | 0 |
| 103/23 | DIRT | 1 | 1 | 2 | 1 | 0 | 0 | 0 |
| 104/23 | DIRT | 2 | 1 | 0 | 0 | 1 | 0 | 0 |
| 105/23 | DIRT | 2 | 2 | 1 | 1 | 1 | 0 | 0 |
| 106/23 | DIRT | 2 | 1 | 1 | 0 | 1 | 0 | 0 |
| 107/23 | MM | 1 | 0 | 1 | 1 | 1 | 1 | 0 |
| 108/23 | DIRT | 2 | 2 | 1 | 1 | 1 | 0 | 0 |
| 109/23 | LTR | 2 | 1 | 0 | 1 | 2 | 0 | 0 |
| 110/23 | DIRT | 1 | 1 | 1 | 1 | 0 | 0 | 0 |
| 111/23 | DIRT | 2 | 2 | 0 | 0 | 1 | 0 | 0 |
| 112/23 | DIRT | 2 | 2 | 0 | 0 | 1 | 0 | 0 |
| 113/23 | MM | 1 | 0 | 1 | 1 | 1 | 1 | 0 |
| 114/23 | DIRT | 2 | 1 | 0 | 1 | 1 | 0 | 0 |
| 115/23 | MM | 1 | 0 | 1 | 0 | 1 | 2 | 0 |
| 116/23 | MM | 1 | 0 | 0 | 0 | 2 | 0 | 0 |
| 117/23 | DIRT | 1 | 0 | 1 | 0 | 0 | 0 | 0 |
| 118/23 | DIRT | 2 | 2 | 0 | 1 | 1 | 0 | 0 |
| 119/23 | DIRT | 2 | 2 | 0 | 0 | 1 | 0 | 0 |
| 120/23 | DIRT | 2 | 1 | 1 | 0 | 1 | 0 | 0 |
| 121/23 | DIRT | 2 | 1 | 2 | 0 | 1 | 0 | 0 |
| 122/23 | DIRT | 2 | 1 | 0 | 0 | 0 | 0 | 1 |
| 123/23 | MM | 1 | 0 | 0 | 2 | 2 | 1 | 0 |
| 124/23 | MM | 1 | 0 | 0 | 1 | 1 | 1 | 1 |
| 125/23 | DIRT | 2 | 1 | 0 | 0 | 0 | 0 | 0 |
| 126/23 | MM | 1 | 0 | 0 | 0 | 1 | 1 | 0 |

The table contain a semiquantitative score based on the following separate histological sub-items:

1. **Fibrous tissue at the base of the fragment**: (0) none, (1) small amount of fibrous tissue at base of the fragment with a clear transition with the trabecular bone, (2) thick fibrous tissue along the entire base of the fragment connected without a clear transition with the trabecular bone.
2. **Neovascularization**: (0) none, (1) 1 or 2 vessels at the base of the fragment, (2) multiple vessels in >1 site of the specimen.
3. **Necrotic fibrocartilaginous tissue**: (0) none, (1) necrotic fibrocartilaginous tissue in < 10% of the entire specimen, (2) necrotic fibrocartilaginous tissue in >10% of the entire specimen.
4. **Necrotic trabeculae**: (0) viable subchondral trabeculae, (1) many osteocyte lacunae are empty, (2) more than a half of the osteocyte lacunae are empty.
5. **Bone resorption**: (0) area deep to the cartilage composed of normal trabeculae and marrow tissues, (1) bone resorption and trabecular bone disconnection in the centre of the specimen, (2) active bone remodelling with proliferation of fibrovascular tissues, new bone formation and resorption.
6. **Degenerated articular cartilage**: (0) normal hyaline cartilage, (1) cartilage with reduced number of chondrocytes and/or sparse chondrocytes cluster, (2) acellular hyaline cartilage, region with fibrillation in the hyaline cartilage and fissures.
